# Identification and Characterization of *Colletotrichum* Species Associated with Maize in Sichuan, China

**DOI:** 10.3390/jof10110799

**Published:** 2024-11-18

**Authors:** Rui Yang, Ying Li, Henan Zhao, Xiaofang Sun, Wen Chen, Pan Li, Xuehu Li, Cuiping Wu, Miaomiao Ma, Guoshu Gong

**Affiliations:** 1 Plan Protection Department, College of Agronomy, Sichuan Agricultural University, Chengdu 611130, China; 2023101018@stu.sicau.edu.cn (R.Y.); ali547721@163.com (Y.L.); hpgwykl@126.com (H.Z.); 2020301154@stu.sicau.edu.cn (W.C.); ngxpxk2023@163.com (P.L.); 2022201067@sicau.edu.cn (X.L.); 71262@sicau.edu.cn (C.W.); 14915@sicau.edu.cn (M.M.); 2 Industrial Crops Research Institute, Sichuan Academy of Agricultural Sciences, Chengdu 610300, China; sunxiaofang207@163.com

**Keywords:** maize anthracnose, *Colletotrichum* species, morphological characterization, phylogenetic analysis, pathogenicity

## Abstract

*Colletotrichum* species are the most common cause of maize anthracnose, which often manifests as leaf spots. However, field observations often reveal symptoms similar to those caused by other leaf spot pathogens, such as *Curvularia* and *Bipolaris*. In this study, 99 isolates were identified using tissue separation and single-spore isolation techniques. As preliminary measures of species diversity, all 99 isolates were identified morphologically, and the glyceraldehyde-3-phosphate dehydrogenase (*GAPDH*) gene sequences were phylogenetically analyzed. Furthermore, 48 representative strains were selected for molecular identification using multi-locus phylogenetic analyses based on five gene loci (ITS, *TUB*, *ACT*, *GAPDH*, and *CAL*). Finally, 10 species of *Colletotrichum* isolated from maize leaf spots were identified. *Colletotrichum cliviicola* was the most dominant species (24.2%), followed by *C. fructicola* (18.2%), *C. karstii* (16.1%), *C. siamense* (13.1%), *C. boninense* (7.1%), *C. kahawae* (7.1%), *C. brevisporum* (6.1%), *C. truncatum* (5.1%), *C. gigasporum* (2.0%), and *C. gloeosporioides* (1.0%). For the first time, pathogenicity tests revealed that *C. cliviicola*, *C. fructicola*, *C. siamense*, *C. karstii*, and *C. truncatum* are the causative agents of maize anthracnose. Additionally, *C. boninense* was identified as an endophytic fungus on healthy maize. In conclusion, this study is the first to identify the pathogen of maize anthracnose in Sichuan Province. It provides valuable insights for accurately diagnosing and managing maize anthracnose.

## 1. Introduction

Maize (*Zea mays* L.), a major food crop, feed, and industrial raw material, is cultivated globally. In China, maize is the most planted crop, covering 43,070 million hectares, with an annual yield reaching 277.203 million tons in 2022 [1].

*Colletotrichum* is among the top 10 plant pathogenic fungi globally [2]. It causes anthracnose in many hosts [3], including vegetables, fruits, and cereals [4,5,6,7,8,9,10,11,12]. According to the U.S. National Fungal Database (https://nt.ars-grin.gov/fungaldatabases/, accessed on 12 November 2024), maize anthracnose can be caused by pathogens such as *C. graminicola*, *C. cereal*, *C. coccodes*, *C. corchori*, *C. gloeosporioides*, *C. sublineola*, and *C. zeae*. The most reported is *C. graminicola* (Ces.) Wils. [13,14]. Maize anthracnose caused by *C. graminicola* is a major disease in maize production, often causing leaf blight and stalk rot and resulting in substantial economic losses [4,15]. Importantly, *C. graminicola* thrives in maize-based agroecosystems, serving as an invasive pathogen of living maize plants and a facultative saprophyte on maize residues [16]. The complete disease cycle accumulates many pathogens in the field and poses a serious threat to maize production. *C. graminicola* has been reported in the United States [4,15], Brazil [17], Portugal [18], Bosnia and Herzegovina [19], and China, etc. [6,20]. It caused severe damage to maize production in the United States [4].

*Colletotrichum* has been dubbed a “catalog of confusion” owing to its ever-changing taxonomy. However, as a model fungus, accurately identifying its species is imperative [3]. In 1852, Cesati identified the fungus isolated from maize and barnyard grass as *Dicladium graminicolum* [21]. In 1914, Wilson renamed the graminicolous *Colletotrichum* species *C. graminicola* owing to its comparable morphology and shared hosts, including maize (*Zea mays* L.), wheat (*Triticum aestivum* L.), and many grasses of the Poaceae family [22]. Von Arx [23] synonymized all 35 graminicolous species as *C. graminicola*, including previously separate taxa such as *C. sublineola*, *C. falcatum*, and *C. caudatum*, based on morphological characteristics including conidia. Using von Arx’s taxonomic system, Sutton concluded that the name *C. graminicola* should only refer to anthracnose on maize based on the morphology and size of conidia and appressoria, pathogenicity, and host range, in accordance with the International Code of Botanical Nomenclature [24,25]. However, cultural characteristics vary with changes in cultural conditions, and *Colletotrichum* species exhibit varied pathogenicity across a wide host range [26]. Notably, many closely related *Colletotrichum* species cannot be distinguished solely based on morphological characteristics [4,23,24,27]. With the advent of molecular biology, the nuclear ribosomal internal transcribed spacer (ITS) region has become the most widely used marker for distinguishing *Colletotrichum* species [28]. For example, isolates from maize are classified as *C. graminicola* based on rDNA ITS-2 sequencing, whereas those from sorghum and *Rottboellia* are identified as *C. subblineolum* [29]. Moriwaki reclassified *Colletotrichum* species in Japan using ITS sequences and morphological characteristics [30]. However, ITS alone is insufficient for differentiating closely related *Colletotrichum* species, such as the *C. gloeosporioides* complex, and many ITS sequences of *Colletotrichum* strains in the National Center for Biotechnology Information (NCBI) GenBank are often erroneous [3,5,26,27,31,32]. Owing to ITS sequence limitations, other markers have been used to classify *Colletotrichum* species. These include the beta-tubulin (*TUB2*), calmodulin (*CAL*), actin (*ACT*), chitin synthase (*CHS-1*), translation elongation factor 1-α (*EF1-α*), glyceraldehyde-3-phosphate dehydrogenase (*GAPDH*), and glutamine synthetase (*GS*) genes [3,31,32,33]. In addition, multi-locus phylogeny has increasingly become a crucial method for classifying and identifying *Colletotrichum*, such as in the *Colletotrichum boninense* complex [34], the *Colletotrichum gloeosporioides* complex [35], and other complex species [36,37,38]. This approach has been consistently used in identifying anthracnose in various fruits, vegetables, and crops, including coffee [5], mango [7], pepper [8], and strawberry [10,11]. Currently, classifying and identifying *Colletotrichum* species requires a combination of morphological characteristics and multi-gene phylogenetic analysis.

Maize diseases are complex, with multiple diseases often affecting maize growth simultaneously, including southern corn leaf blight (SCLB, *Bipolaris maydis*), northern corn leaf blight (NCLB, *Exserohilum turcicum*), maize gray leaf spot (*Cercospora zeae-maydis*), and *Curvularia* leaf spot (*Curvularia lunata*). The conducive humidity and temperature in Sichuan provide a favorable environment for the development of these maize diseases [39,40]. Consequently, we conducted an extensive survey and sample collection to identify the pathogenic species responsible for maize leaf spot disease in Sichuan Province. During our study, *Colletotrichum* species were frequently isolated. Given the lack of reports on maize anthracnose in Sichuan Province, we considered the *Colletotrichum* species we collected, their pathogenicity, and whether maize anthracnose occurs concurrently with other leaf spot diseases. The field symptoms of leaf spot diseases closely resembled those of anthracnose, making them challenging to differentiate.

Globally, the predominant cause of maize anthracnose is attributed to *C. graminicola*. While *C. graminicola* has been identified as the causal agent of anthracnose leaf blight on maize in China [20], further investigation is needed regarding maize anthracnose in Sichuan Province, China. Furthermore, clarification is needed on whether all species isolated from maize exhibit similar pathogenicity and host specificity. Therefore, this study aimed to identify the *Colletotrichum* species associated with maize leaf spots in Sichuan by combining morphological characteristics with multi-locus phylogenetic analysis, providing a more scientifically grounded approach for the diagnosis and management of maize leaf blight.

## 2. Materials and Methods

### 2.1. Sample Collection and Fungal Isolation

During the growing season, we collected samples of maize leaf spots with visible and diverse lesions from 20 localities across 12 central municipal districts in the maize-producing areas of Sichuan Province (Table S1). Tissues (5 × 5 mm) were taken from the edges of lesions and surface-sterilized with 75% ethanol (*v*/*v*) for 30 s, followed by 1% NaOCl (*w*/*v*) for 10 s. They were washed three times with sterilized distilled water. The treated tissues were placed on sterile potato dextrose agar (PDA) disks containing streptomycin (30 µg/mL) and incubated at 25 °C in the dark for seven days [11,41]. Single-spore cultures were obtained from all *Colletotrichum* isolates following the method described by Gong et al. [42] A single fungal spore was aseptically inoculated onto a water agar block using sterilized acupuncture needles. This block was then transferred to fresh culture plates and incubated at a controlled temperature of 25 °C for 3 days. Later, the hyphal block was transferred to PDA plates and incubated at 25 °C for 5 days. Finally, one isolate per lesion was maintained on PDA slants at 4 °C for short-term storage and in 50% glycerol at −80 °C for long-term storage.

### 2.2. Morphological and Cultural Characterization

All isolates were cultured on PDA and incubated in the dark for 5–7 days at 25 °C. Colony diameter (length and width of the colony) was measured daily using the crossover method until the plate was entirely covered. The daily growth rate was estimated using values from three replicates. Meanwhile, colony color, size, and shape were recorded. The lengths and widths of conidia, conidia appressoria, and mycelial appressoria were measured for 30 conidia per isolate. To generate conidial appressoria, conidia were inoculated in sterile distilled water on a microscope slide, which was subsequently placed in a Petri dish with moistened absorbent paper and incubated at 25 °C in darkness. The morphological characteristics of conidial appressoria were observed and recorded using a compound microscope after 24 h. Mycelial appressoria were generated using a modified slide culture method. A 2 × 2 cm block of water agar (WA) was placed on a sterile slide. A 3- to 4-day-old mycelium was extracted from the edge of the colony and attached to the edge of the WA. The slide was covered with a sterile coverslip, placed in a sterile Petri dish, and then incubated for seven days in the dark [8,24,26]. Samples for microscopy were prepared using clear water and observed with a compound microscope (Axio Imager Z2, Carl Zeiss, Oberkochen, Germany). Data on the radial colony growth rate, conidial size, and conidial appressoria size were evaluated statistically using the Statistical Package for the Social Sciences software (SPSS, version 21.0).

### 2.3. DNA Extraction

After incubating all isolates on PDA for seven days at 25 °C, mycelia were scraped off using sterile spoons and transferred to 1.5 mL centrifuge tubes containing liquid nitrogen. The mycelia were ground into powder using a sterile grinding rod. Genomic DNA from each isolate was extracted using the Ezup Column Fungi Genomic DNA Purification Kit (Sangon Biotech, Shanghai, China) following the manufacturer’s instructions. DNA concentration and quality were assessed using a Thermo Scientific NanoDrop™ 2000 Spectrophotometer (Thermo Fisher Scientific, Wilmington, DE, USA).

### 2.4. Polymerase Chain Reaction (PCR) Amplification and Sequencing

PCR amplification was performed using an S1000TM Thermal Cycler (Bio-Rad Laboratories, Feldkirchen, Germany). *GAPDH* was amplified for all isolates using GDF/GDR primers (Table 1) [7,43]. Subsequently, *ACT*, *TUB2*, *CAL*, and ITS were amplified for representative strains from different clades using the following primers: ACT512F/ACT783R [44], Bt2a/Bt2b [45], CL1/CL2A [46], and ITS1/ITS4 [47,48], respectively. PCR was performed under the conditions described by Liu [8]. Amplification was performed in a 25 μL mixture containing 10.5 μL ddH_2_O, 12.5 μL 2× PCR MasterMix (Sangon Biotech Co., Ltd., China), 0.5 µL upstream primers (10 µM), 0.5 µL downstream primers (10 µM), and 1 µL DNA templates (~100 ng/µL). The PCR-amplified products were visualized on a 1% agarose gel and sequenced by Shanghai Biological Engineering Co., Ltd., Shanghai, China.

### 2.5. Phylogenetic Analyses

Phylogenetic analysis was conducted as previously described [8,11]. BLASTn was used to compare the retrieved sequences on the NCBI database with reference sequences for *ACT*, *TUB2*, *CAL*, *GAPDH*, and the ITS region from GenBank. The sequences of 99 representative isolates and 40 references were edited using Chromas (version 2.3), with manual adjustments made when necessary. All sequences were aligned using Clustal X (version 2.0) [43,49]. A neighbor-joining (NJ) tree based on the *GAPDH* gene of 99 isolates was constructed using Molecular Evolutionary Genetics Analysis (MEGA) (version 7.0) [50] to preliminarily group and select representative isolates for multi-locus phylogenetic analysis. Concurrently, distinct phylogenetic trees were constructed using the NJ method to compare the differences in phylogenetic analyses of single-locus genes.

In addition, maximum likelihood (ML) and Bayesian inference (BI) trees were constructed from concatenated sequences of the five gene fragments (*ACT*-*TUB2*-*CAL*-*GAPDH*-ITS) using PhyloSuite software [51]. BI phylogenies were inferred using MrBayes (version 3.2.6) [52] under the partition model (2 parallel runs and 5,000,000 generations), with the initial 25% of sampled data discarded as burn-in. ML phylogenies were inferred using IQ-TREE [53] under the edge-linked partition model with 1000 standard bootstraps and the Shimodaira–Hasegawa-like approximate likelihood ratio test [54]. ModelFinder [55] was used to select the best-fit partition model for BI (edge-linked) using the Bayesian information criterion (BIC) and ML using the corrected Akaike information criterion (AICc). Finally, five gene substitution models were selected: *ACT* and *TUB2*: HKY+F+G4, *CAL*: K2P+I, *GAPDH*: K2P, ITS: SYM+G4 based on BIC; *ACT* and *TUB2*: TIM2+F+I+G4, *CAL*: TIM3+F+I+G4, *GAPDH*: TNe, and ITS: TIM+F+G4 based on AICc.

Moreover, a maximum parsimony (MP) tree was constructed using Phylogenetic Analysis Using Parsimony (PAUP) (version 4.0 b10) (Swofford, 2002) [56] with a heuristic search option, 1000 random sequence additions, and *Monilochaetes infuscans* (CBS 869.96) as the outgroup from GenBank (Table S2). All gaps were treated as missing data, and the maximum number of trees was unlimited, with zero-length branches collapsed. All multiple parsimonious trees were saved, and clade stability was assessed using a bootstrap analysis with 1000 replicates. Afterward, the tree length (TL), consistency index (CI), retention index (RI), related consistency index (RC), and homoplasy index (HI) were calculated [56].

### 2.6. Pathogenicity Test

Pathogenicity tests were performed on maize leaves of potted seedlings and field-grown maize using both non-wound and wound inoculation methods as previously described [7,11,40].

Based on the results of morphological identification and phylogenetic analysis, strain selection for each species considered morphological characteristics, geographical origins, phylogenetic branch placements, and other subtle differences. A representative set of strains was selected for each species, with five strains chosen (select all available strains if fewer than five) as the test strains. In total, 43 representative strains underwent pathogenicity testing to determine whether all species were pathogenic to maize. The maize variety Zhongyu 3, widely planted in Sichuan’s main maize-producing areas, was selected for the pathogenicity test. Strains were revitalized on PDA and incubated for 7–10 days at 25 °C in darkness until adequate sporulation occurred. To establish a standard inoculum for pathogenicity, conidia were suspended in sterile distilled water containing 0.01% (*v*/*v*) Tween 80, with a concentration of 1 × 10^6^ conidia/mL. The conidial suspension was filtered through two layers of muslin cloth.

Wound and non-wound inoculations were performed on potted seedlings and leaves of maize grown in vivo. Potted maize was inoculated when it reached the 3–4 leaf stage. In the wound inoculation process, each location was wounded with a sterilized acupuncture needle (0.25 mm diameter). Leaves of potted maize and those of field-grown maize in vivo were inoculated with the conidial suspension, with 3–5 replicates per leaf depending on leaf size and applying 50 μL of the suspension at each location. Concurrently, non-wounding inoculation was performed on potted maize in vivo, with approximately 5 mL of the suspension being uniformly sprayed onto each leaf. Following inoculation, maize leaves were covered with plastic film to maintain moisture for 24 h at a relative humidity of 90%, after which the plastic film was removed. The potted seedlings after inoculation were placed in a greenhouse at 25 °C with a 12 h day/night regime and monitored daily for lesion development. Leaves inoculated with sterile distilled water containing 0.01% (*v*/*v*) Tween 80 served as controls. Inoculations in the field were conducted when the maize plants reached the 8-leaf stage. The inoculated leaves were incubated using the method mentioned above, maintaining moisture for 24 h. It was cloudy during inoculation in the field, with temperatures ranging from 18 to 29 °C and humidity around 60–70%.

All leaves used for inoculation were washed and surface-sterilized with 75% ethanol for 1 min, rinsed three times with sterile distilled water, and then air-dried. The experiments were repeated three times. The formation of typical necrotic lesions was considered a successful infection. Disease incidence was recorded following inoculation.

### 2.7. Host Specificity Test

To determine anthracnose host specificity, one representative strain from each species was used to inoculate Crown Pear (*Pyrus bretschneideri* Rehd.) and Red Fuji Apple (*Malus domestica* Borkh. CV. Red Fuji) using conidial suspension [8,40]. The pears and apples were washed, surface-sterilized with 75% ethanol for 1 min, rinsed three times with sterile distilled water, and then air-dried. Subsequently, 50 μL of conidial suspension was inoculated onto the surface of the fruits, which had been slightly wounded with a sterilized acupuncture needle. To maintain humidity, the inoculated fruits were covered with plastic film and incubated for seven days in a growth chamber at 25 °C with a 12 h light/12 h dark photoperiod. Lesion diameters were measured using the cross method to assess virulence. All experiments were repeated three times.

Following Koch’s postulates, all fungal strains used in the pathogenicity tests were re-isolated to confirm their identity via the molecular and morphological methods previously described.

### 2.8. Data Analysis

Pathogenicity test data and morphological characteristics are presented as mean ± standard deviation. Statistical analysis was performed using a one-way analysis of variance followed by Duncan’s new multiple-range test to determine significant differences. Values labeled with different letters (a, b, or c) indicate significant differences at the 0.05 level, as determined using SPSS (version 24.0, SPSS Inc., Chicago, IL, USA).

## 3. Results

### 3.1. Morphological and Cultural Characteristics

**Figure 1:**
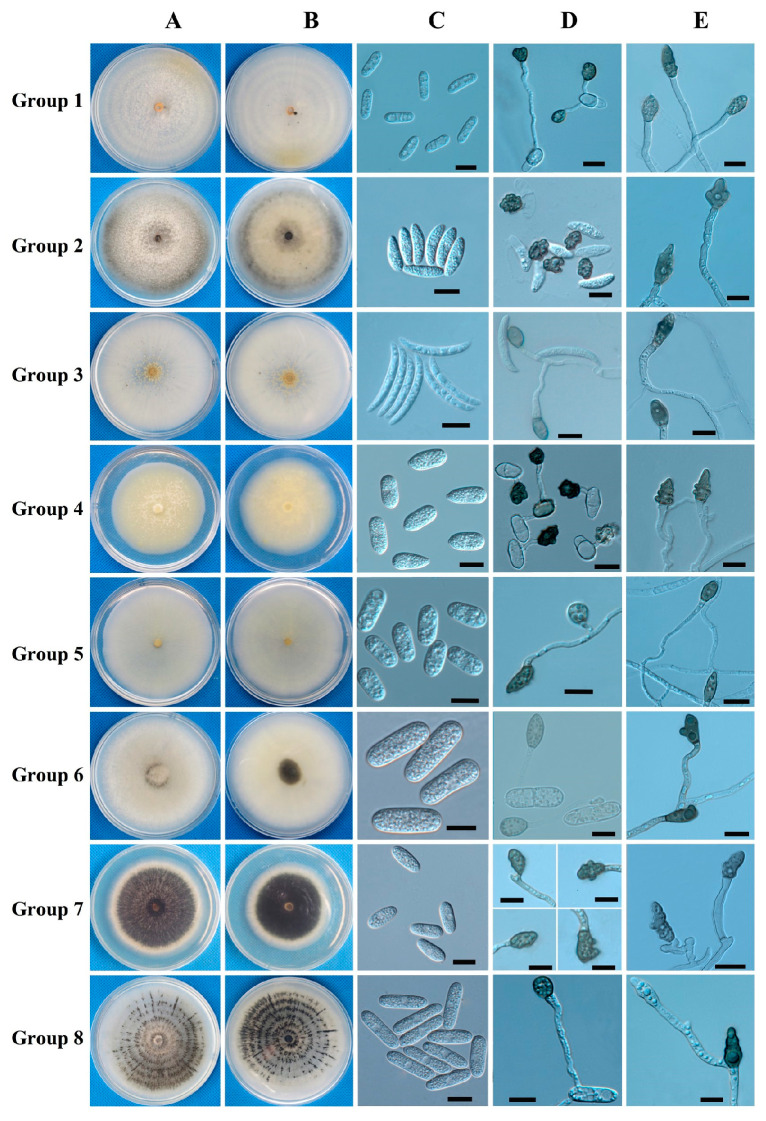
Morphological and cultural characterization of *Colletotrichum* spp. on maize. The characterization was carried out with isolates incubated on potato dextrose agar (PDA) at 25 °C in the dark for 5–7 days. (**A**,**B**), Above and below of colonies on PDA; (**C**), conidia; (**D**), conidial appressoria; (**E**), mycelial appressoria; scale bars = 10 μm.

Following tissue separation and single-spore isolation, we obtained 99 isolates, which were classified into eight groups based on morphological characteristics (Figure 1, Table 2 and Table S1). Group 1 included 32 isolates consistent with the description of the *C. gloeosporioides* complex. Group 2 comprised 24 isolates matching the description of *C. cliviicola.* Group 3 comprised five isolates fitting the description of *C. truncatum*; Group 4 included seven isolates corresponding to *C. boninense*; and Groups 5 and 6 included sixteen and two isolates, respectively, matching the characteristics of *C. karstii* and *C. gigasporum*. Moreover, seven isolates belonged to Group 7, matching the description of *C*. *kahawae*. Group 8 comprised six isolates matching the description of *C*. *brevisporum*. Table 2 and Figure 1 provide an overview of the morphological data for these *Colletotrichum* species from Groups 1 to 8.

#### 3.1.1. Colony Characteristics

Each group exhibited a unique morphology on PDA after seven days. Group 1 isolates developed pale white colonies with short white aerial mycelia. The reverse side of the colonies was white, and numerous bright orange conidial masses were observed near the inoculum point. Group 2 isolates developed colonies ranging from gray to black, with aerial mycelia that initially appeared white and later turned gray-black. Group 3 isolates developed conidial masses varying from pale yellow to black-gray, accompanied by sparse white aerial mycelia. Group 4 isolates exhibited pale yellow colonies with flocculent and rare aerial mycelia. Group 5 colonies were white with a slight yellowish tint, displaying sparse aerial mycelia and few conidial masses. Group 6 isolates developed dense white colonies with a small amount of gray aerial mycelium. Group 7 colonies demonstrated a clear outline with distinct black, cream, and white stratification, whereas Group 8 colonies exhibited radial and concentric black streaks with a few gray aerial mycelia (Figure 1A,B).

#### 3.1.2. Growth Rate

The growth rate varied significantly among the different groups. Group 1 isolates exhibited the highest mycelial growth rate at 5.9 ± 0.3 mm/day, followed by Group 2 at 5.5 ± 0.2 mm/day. Group 4 displayed a growth rate of 4.9 ± 0.2 mm/day, followed by Group 8 at 4.8 ± 0.1 mm/day and Group 6 at 4.7 ± 0.2 mm/day. Group 7 displayed a growth rate of 4.6 ± 0.4 mm/day, and Groups 5 and 3 exhibited growth rates of 4.6 ± 0.2 and 4.5 ± 0.4 mm/day, respectively (Table 2).

#### 3.1.3. Conidial Morphology

Five different conidial types were observed across the eight groups, each with a distinct shape: cylindrical (observed in Groups 1, 5, and 7), fusiform (observed in Group 2), falcate (observed in Group 3), taper (observed in Group 4), and long cylindrical (observed in Groups 6 and 8). The conidia in Group 5 exhibited shorter lengths than those in the other groups. Groups 6 and 8 demonstrated long cylindrical conidial shapes; the conidia in Group 6 were considerably wider and ranked second in length among all groups, significantly different from those in Group 8. The conidia in Group 3 were the longest, sickle-shaped, gradually tapering toward the ends, and distinctly different from those in other groups. The conidia in Group 4 were tapered, with bluntly rounded and slightly pointed ends. Except for Groups 3 and 4, the conidia were generally cylindrical, making individual identification difficult (Figure 1C).

#### 3.1.4. Conidial Appressorium Morphology

Conidial appressoria can be classified into three categories based on shape and color. The first category included Groups 3, 5, 6, and 7, characterized by ovoid and brown appressoria. In Groups 3 and 6, the edges of the appressoria were smooth, whereas those in Groups 5 and 7 were slightly dentate. The second category comprised Groups 1 and 8, where the appressoria were suborbicular or subelliptic and dark brown. The third category included Groups 2 and 4, which displayed irregular shapes with crenate edges and were dark brown (Figure 1D). Significant differences were observed in appressorial length and width among all groups. Group 6 exhibited the longest and widest appressorium, whereas Group 1 exhibited the shortest, measuring approximately half the length of that in Group 6 (Table 2).

#### 3.1.5. Mycelial Appressorium Morphology

The mycelial appressoria produced by the isolates varied from ovoid to slightly irregular and irregular, with colors ranging from light to dark brown. Appressoria in Groups 1, 3, 5, and 8 displayed shallow or deep cracks around the edges and were more regular. Conversely, the edges of the remaining groups displayed more protrusions, shallow or deep clefts, and diverse morphologies (Figure 1E). The length of the mycelial appressoria varied substantially between the groups, whereas width differences were non-significant. Group 8 exhibited the longest mycelial appressoria, measuring 16.5 ± 1.4 μm, and Group 1 exhibited the shortest, measuring only 9.9 ± 2.2 μm (Table 2).

### 3.2. Phylogenetic Analysis

**Figure 2:**
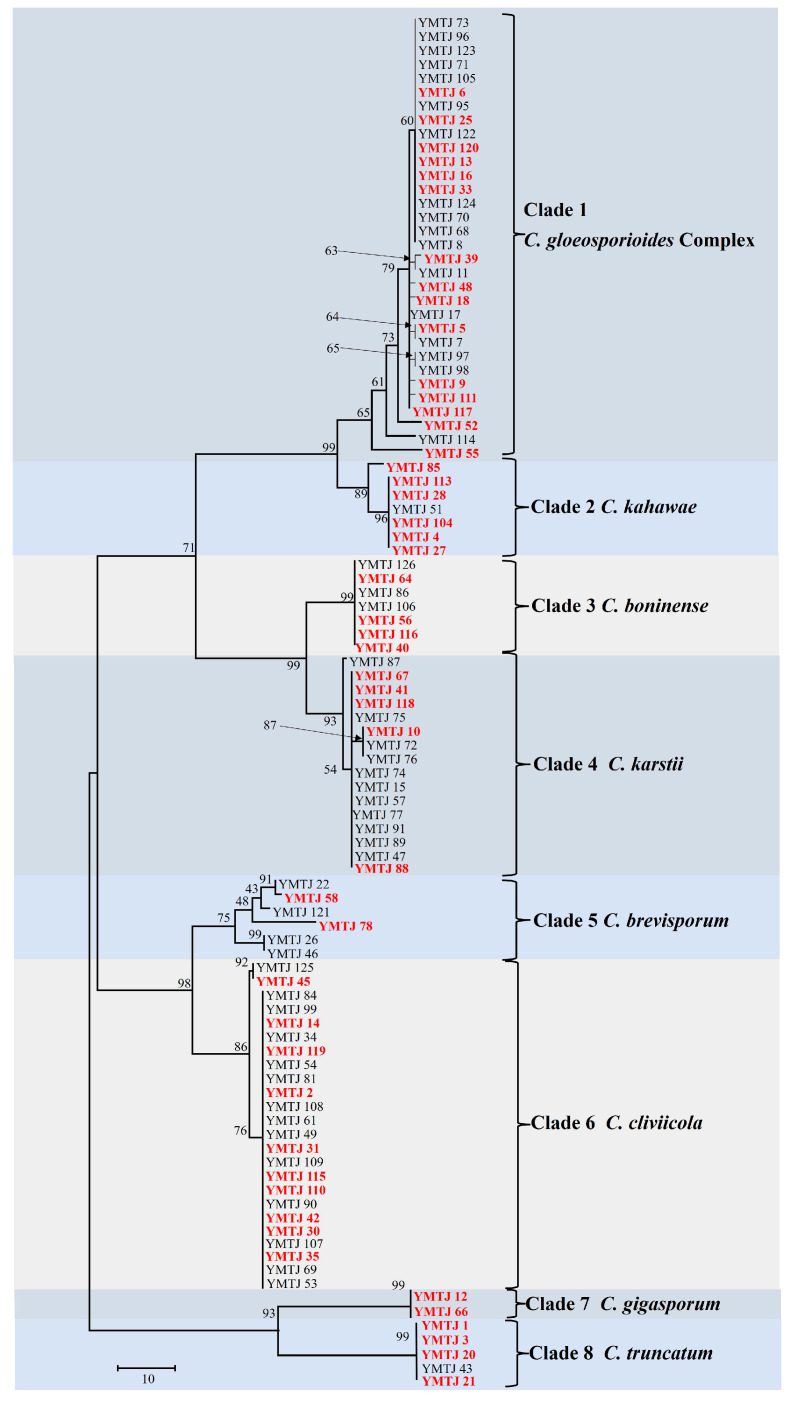
Neighbor-joining tree based on *GAPDH* gene sequences of 99 *Colletotrichum* isolates. Isolates selected for subsequent phylogenetic analyses are highlighted in red.

**Figure 3:**
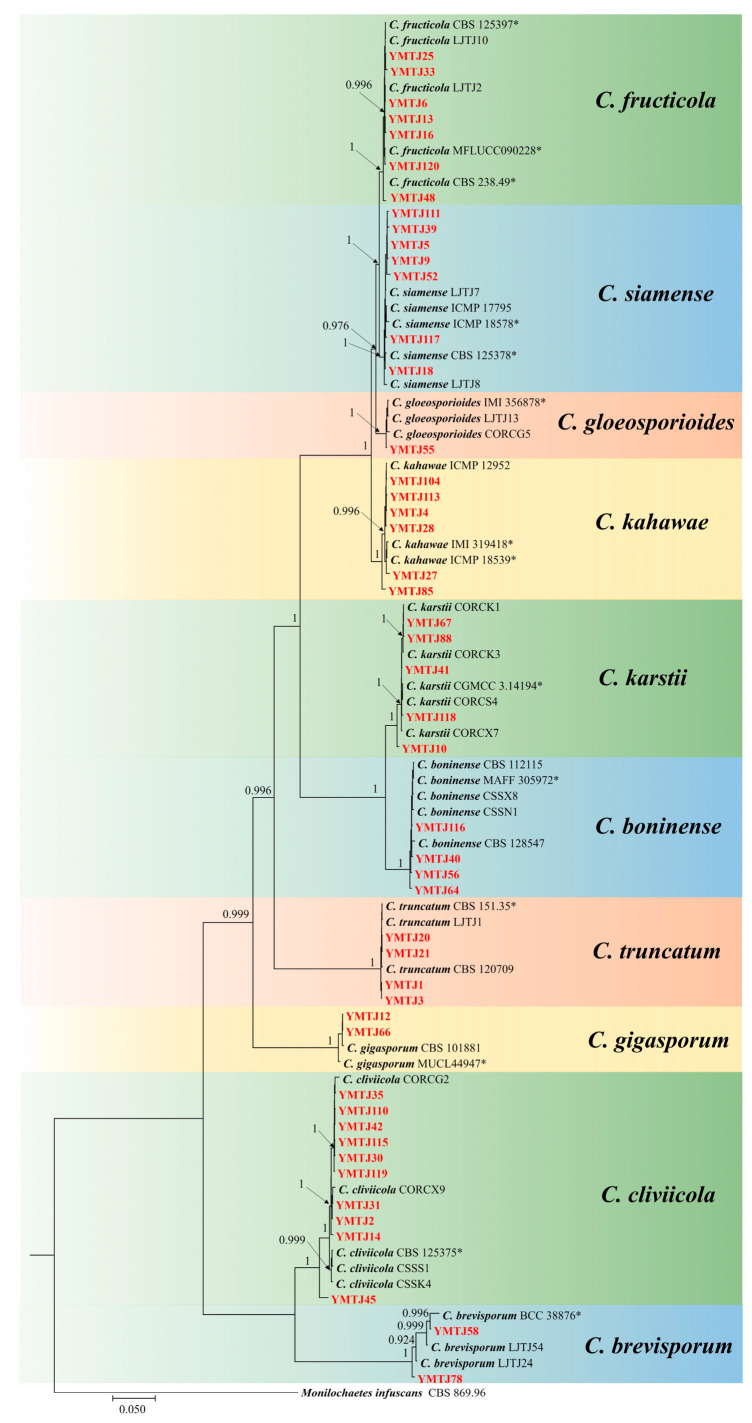
A Bayesian inference species tree of the *Colletotrichum* species. Phylogram generated based on concatenated sequences of *ACT*, *TUB2*, *CAL*, *GAPDH*, and ITS genes each with a separate model of DNA evolution, showing the phylogenetic relationships of *Colletotrichum* species associated with maize leaves from Sichuan Province, China. Bayesian posterior probability (PP ≥ 0.90). Isolates from this study are shown in red and bold. The tree is rooted with *Monilochaetes infuscans*.

Based on the phylogenetic analysis of the *GAPDH* gene, all 99 isolates were classified into eight major clades (Figure 2 and Table S1). Each single-locus gene was capable of distinguishing the species within the complex. Phylogenetic analysis, including reference strains, allows for the preliminary differentiation of *C. gloeosporioides* within its complex. Other species are distributed across distinct branches, achieving a preliminary classification. The *CAL* gene differentiated between *C. simense* and *C. fructicola* within the *C. gloeosporioides* species complex (Figure S1). From these clades, 48 representative strains were selected for multi-locus phylogenetic analysis, including the *C. gloeosporioides* complex, *C. cliviicola*, *C. truncatum*, *C. boninense*, *C. karstii*, *C. gigasporum*, *C. kahawae*, and *C. brevisporum*, with *Monilochaetes infuscans* (CBS 869.96) serving as an outgroup. Based on MP, the dataset for the five genes (*ACT*, *TUB2*, *CAL*, *GAPDH*, and ITS) comprised 1610 characters, including alignment gaps, with 877 constant, 612 parsimony-informative, and 121 parsimony-uninformative. The parsimony analysis yielded the most parsimonious tree (TL = 1505, CI = 0.7176, RI = 0.9671, RC = 0.6940, and HI = 0.2824). Subsequent BI (Figure 3), ML (Figure S2), and MP tree (Figure S3) constructions revealed that 48 *Colletotrichum* strains belonged to 10 distinct clades. Clade 1 isolates were classified into three distinct clades: *C. gloeosporioides*, *C. simense*, and *C. fructicola*. Phylogenetic results indicated that *C. fructicola*, *C. simense*, *C. gloeosporioides*, and *C. kahawae* belong to the *C. gloeosporioides* species complex; *C. boninense* and *C. karstii* belong to the *C. boninense* species complex; and *C. cliviicola* and *C. brevisporum* belong to the *C. orchidearum* and *C. magnum* species complexes, respectively. Moreover, combining single-gene and multi-gene phylogenetic analyses with morphological characteristics, all of the 99 isolates were classified into 10 species: *C. cliviicola* (24 isolates, 24.2%), *C. fructicola* (18 isolates, 18.2%), *C. karstii* (16 isolates, 16.1%), *C. simense* (13 isolates, 13.1%), *C. boninense* (7 isolates, 7.1%), *C. kahawae* (7 isolates, 7.1%), *C. brevisporum* (6 isolates, 6.1%), *C. truncatum* (5 isolates, 5.1%), *C. gigasporum* (2 isolates, 2.0%), and *C. gloeosporioides* (1 isolate, 1.0%).

**Figure 4:**
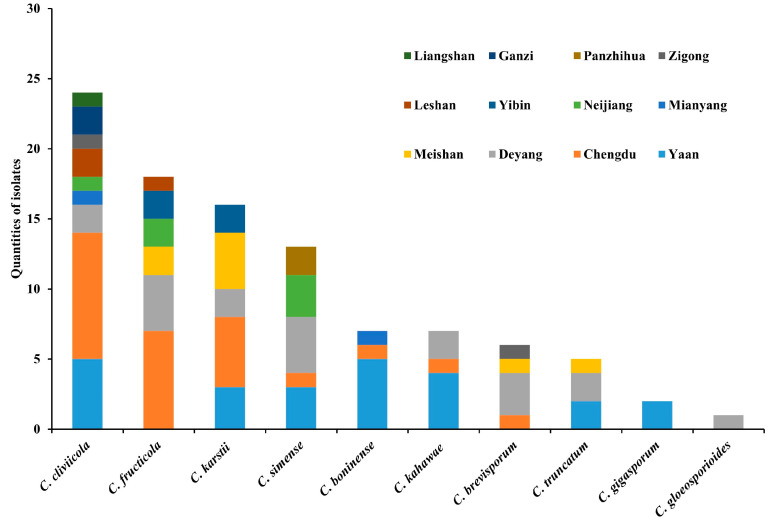
Distribution of *Colletotrichum* species in different maize-producing localities of Sichuan.

*Colletotrichum cliviicola*, obtained from nine collection areas, was the most predominant species in the primary maize-producing regions of Sichuan Province. *C. gigasporum* and *C. gloeosporioides* were collected from one collection area each, indicating their rarity in Sichuan Province (Figure 4). The most frequently reported maize anthracnose pathogen (*C. graminicola*) was absent among the identified isolates.

### 3.3. Pathogenicity

**Figure 5:**
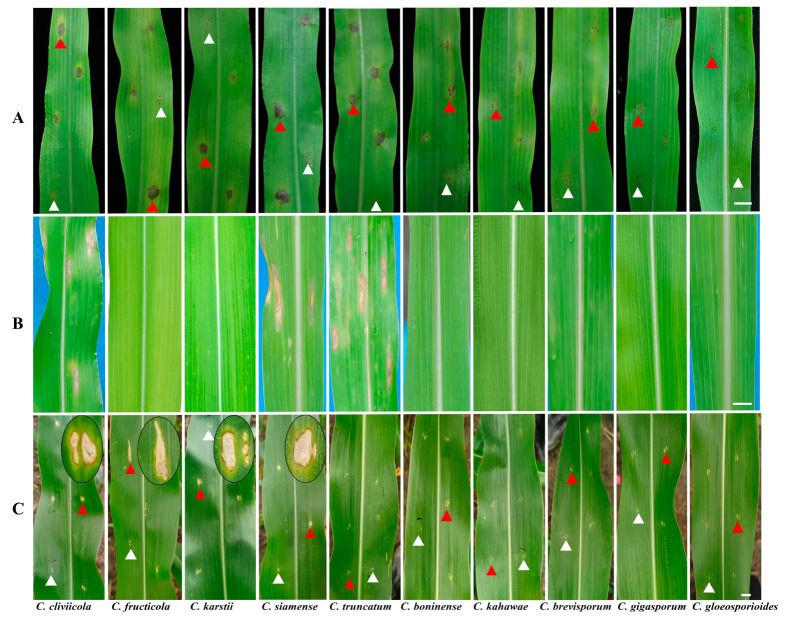
Symptoms caused by inoculation of representative strains of *Colletotrichum* species on potted seedlings and field-grown maize leaves through wound inoculation methods of variety Zhongyu 3. (**A**) Potted seedlings via wound inoculation; (**B**) potted seedlings via non-wound inoculation; (**C**) field-grown via wound inoculation. The red triangles represent symptoms of maize anthracnose after inoculation, and the white triangles represent blank controls inoculated with sterile distilled water with 0.01% (*vol*/*vol*) Tween 80. The observed symptoms on the fifth to seventh day after inoculation. Scale bar = 1 cm.

Through wound inoculation, we found that strains of five species (*C. cliviicola*, *C. fructicola*, *C. karstii*, *C. siamense*, and *C. truncatum*) caused distinct dark brown lesions with chlorosis and sub-fusiform shapes. *C. cliviicola* induced light brownish lesions with pronounced chlorosis, while *C. fructicola* lesions were relatively small and brown. *C. karstii* caused light brown lesions with surrounding chlorosis in a spindle shape. *C. siamense* caused the largest and most distinct dark brown necrotic lesions, while *C. truncatum* induced dark brown lesions with smaller chlorotic halos. *C. boninense* resulted in minor light brown lesions accompanied by chlorosis symptoms, whereas *C. kahawae* and *C. brevisporum* showed only a few areas of chlorosis and no necrotic spots. The fungus could not be re-isolated. *C. gigasporum* and *C. gloeosporioides* caused almost no chlorosis or necrosis, and no lesions were present in all the control treatments (Figure 5A). Inoculation with a non-wounding spray produced divergent outcomes. By the third day following inoculation, the leaves exhibited distinct grayish-white circular necrotic lesions as a result of infection by three species (*C. cliviicola*, *C. siamense*, and *C. truncatum*). On the seventh day after inoculation, the lesions had developed into streaked or irregular shapes, with some exhibiting visible concentric rings. The centers of the lesions were grayish-white, surrounded by a light brown to gray-black margin. Among them, *C. siamense* formed the longest lesions. The remaining seven species did not produce any lesions, similar to the control treatments, (Figure 5B).

In the field trial, strains of *C. cliviicola*, *C. fructicola*, *C. siamense*, and *C. karstii* resulted in grayish-white irregularly shaped lesions near the inoculation sites, characterized by brownish edges and areas of chlorosis. Notably, lesions caused by *C. fructicola* expanded into elongated strips. In contrast, the remaining species—*C. truncatum*, *C. boninense*, *C. kahawae*, *C. brevisporum*, *C. gigasporum*, and *C. gloeosporioides*—failed to induce any lesions (Figure 5C).

Table 3 depicts the incidence of the different *Colletotrichum* species on maize leaves in potted seedlings and field-grown plants. Generally, the disease incidence rate following wounding inoculation was higher compared to non-wounding inoculation. Furthermore, the success rate of inoculation of potted seedlings in the greenhouse was greater than that observed under field conditions. In three conditions, the dominant species—*C. cliviicola*—infected maize leaves. However, the success rates for non-wounding and field inoculation were comparatively lower, at 38.9% and 33.3%, respectively. And the non-wounding inoculation of the sub-dominant species *C. fructicola* was unsuccessful. From the results of the three inoculations, it can be inferred that *C. siamense* possesses the highest virulence, which exhibited a 72.2% success rate with potted seedlings via non-wounding inoculation and 53.3% in the field. However, *C. karstii* had a relatively low infection success rate in potted seedlings, with an even lower rate in the field, approximately 16.7%, and non-wounding inoculation was unsuccessful. *C. truncatum* successfully infected potted seedlings but failed in the field-grown maize leaves. The remaining species—*C. boninense*, *C. kahawae*, *C. brevisporum*, *C. gigasporum*, and *C. gloeosporioides*—demonstrated a 0% success rate in all three conditions.

Therefore, based on the aforementioned results, we conclude that *C. cliviicola*, *C. fructicola*, *C. siamense*, *C. karstii*, and *C. truncatum* are the causative agents of maize anthracnose.

### 3.4. Host Specificity

**Figure 6:**
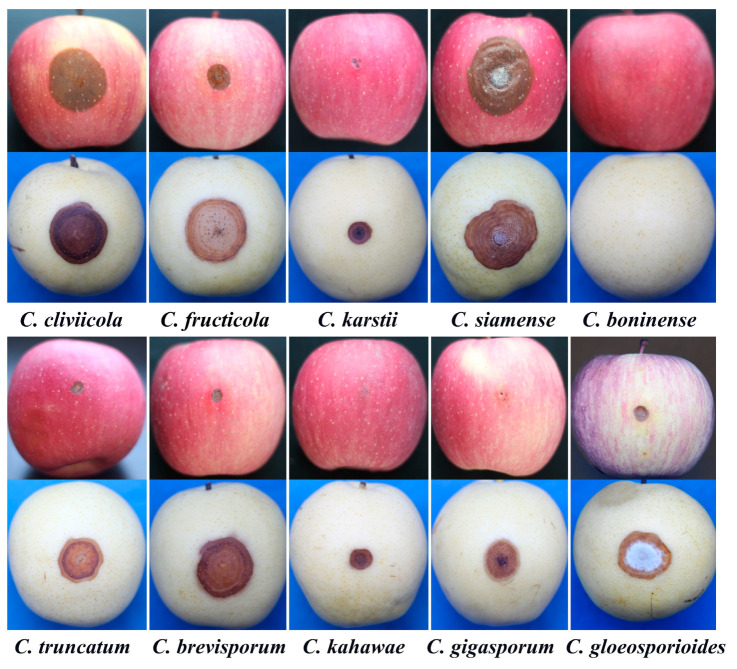
Symptoms on Crown Pear and Red Fuji Apple inoculated by all ten representative *Colletotrichum* isolates. The observed symptoms on the fifth to seventh day after inoculation.

Some species of *Colletotrichum* caused disease in Crown Pears and Red Fuji Apples three days after inoculation. However, disease development differed. Brown verticillate lesions were observed on Crown Pear seven days after inoculation, with the exception of *C. boninense* (Figure 6). The lesion diameters ranged from 0.8 to 4.4 cm. *C. siamense* generated lesions up to 4.4 ± 0.7 cm in diameter, significantly larger than those caused by other species, indicating its strong pathogenicity. Only six species (*C. cliviicola*, *C. fructicola*, *C. karstii*, *C. siamense*, *C. truncatum,* and *C. gloeosporioides*) caused lesions on Red Fuji Apples. These lesions appeared as brown water-stained spots on the apple surface, with orange-red conidial masses and white mycelium development. The lesion diameters ranged from 0.5 to 3.5 cm. Additionally, *C. siamense* exhibited strong pathogenicity on Red Fuji Apples (Table 4).

The pathogenicity tests confirmed Koch’s postulate, with re-isolated strains identified based on morphological characteristics and *GAPDH* sequence.

Based on extensive sampling, isolation, and identification over several years (Table S3), as well as several pathogenicity assays, we identified *C. cliviicola*, *C. fructicola*, *C. siamense*, *C. karstii*, and *C. truncatum* as the causal species of maize anthracnose. In addition, we discovered that *C. boninense* is an endophyte, whereas *C. cliviicola* and *C. fructicola* are the primary causal species, covering 85.0% of the sampling area in Sichuan Province. Furthermore, *C. siamense* emerged as a potent pathogen capable of inducing leaf necrosis across different maize varieties. This study is the first report on maize anthracnose and the initial identification of its pathogenic fungi in Sichuan. Except for *C. boninense*, all other non-host-specific species demonstrated pathogenicity toward maize, Crown Pears, and Red Fuji Apples.

## 4. Discussion

Maize is not only a staple cereal crop in Sichuan Province but also an essential raw material for feed and industry [39]. The genus *Colletotrichum*, encompassing a range of plant pathogens including those affecting both economically significant and staple crops, holds substantial importance in agricultural production and scientific research due to the severity of the losses it inflicts and its distinctive modes of life (endophytic, biotrophic, and hemibiotrophic lifestyles) [57]. To effectively prevent and treat diseases caused by multiple pathogens, the pathogens in each disease triangle should be managed; otherwise, the diseases will spread further [58].

Due to historical limitations, morphological identification and host specificity have traditionally dominated the taxonomy of *Colletotrichum* species [27,28]. Consequently, morphological identification serves as a significant basis for species classification. For example, *C. gigasporum* produces large conidia (>20 μm), and *C. truncatum* forms sickle-shaped conidia, which are distinctive features that differentiate their complex species from others [59]. In our study, the morphological characteristics of *C. gigasporum*, *C. kahawae*, and *C. truncatum* are consistent with the fundamental descriptions provided by previous reports [8,35,60,61,62]. Those of *C. boninense* and *C. karstii* are identical; however, compared to previous studies, the conidia of *C. boninense* in our study are longer than *C. karstii* [32,34]. The morphological characteristics of *C. brevisporum* follow those described by Liu et al. [8], but the conidia and appressoria are larger than the strain isolated from *Anthurium* sp. (culture CBS 129957) [38,63]. The conidial color and size of *C. cliviicola*, as well as the morphology of the appressoria, are similar to those from *Clivia miniata* (ex-holotype culture CBS 125375) [38]. However, there are morphological differences in the conidia. The strain isolated from maize exhibits conidia that taper at both ends. The *C. gloeosporioides* species complex contains three species, *C. gloeosporioides*, *C. simense*, and *C. fructicola*, whereas Liu et al. distinguished *C. fructicola* from the complex [8]. Furthermore, Weir et al. [35] describe distinct morphological differences among the three species, possibly due to their origins from different countries or hosts. However, the morphology of the conidia is in agreement with their findings.

In brief, morphological identification can be recorded as characteristic of species, but it should not be considered a reliable definitive criterion. For instance, *C. siamense* strains ICMP 18618 and ICMP 18578, originating from different hosts within the same country, exhibit different pure culture colony morphologies, manifesting as gray-brown zonate and pale orange, respectively [35]. Therefore, morphological traits constitute the initial step in the identification of species. It is an essential taxonomic characteristic for differentiating species complexes. It is imperative to pay attention to subtle differences under consistent cultural conditions.

Phylogenetic analysis has significantly expanded our understanding of species diversity and is the most crucial and essential method for species identification [57]. However, the performance of single genes in resolving species is different. *C. cliviicola* can be effectively distinguished from its closely related species, *C. plurivorum*, based on the *TUB2*, *HIS*, and *GAPDH* genes, whereas ITS and *ACT* are ineffective for this purpose [38]. *GAPDH* and *TUB* differentiate *C. brevisporum* and *C. truncatum* from their respective complexes, respectively [3,59]. Based on the results of Liu et al. [33], sequencing of the *CAL*, *ApMat*, and *GS* loci is necessary to ascertain *C. kahawae*. The *C. gloeosporioides* species can be differentiated using the ITS region, which aligns with our single-gene phylogenetic analysis; however, the ITS region is ineffective in distinguishing between *C. simense* and *C. fructicola*. In contrast, the *CAL* gene can successfully resolve these two species, which is consistent with Weir’s findings [35].

*C. karstii* and *C. boninense* are members of the *C. boninense* species complex, and all species within this complex can be distinguished using *GAPDH* as a single marker [57]. In our study, we successfully achieved preliminary discrimination among species by utilizing five different genes. This may be attributed to the fact that we categorized our species into six distinct complexes: Gloeosporioides (four species), Boninense (two species), Truncatum (one species), Gigasporum (one species), Orchidearum (one species), and Magnum (one species). However, it is also possible that this finding is influenced by the limited number of species we included in our analysis.

Furthermore, our results show a high level of concordance between single- and multi-gene phylogenetic analyses. The species classification, from the main trunk to the most terminal branches, is broadly consistent with the latest research [57,59]. The branches resolved by different methods in multi-gene phylogenetic analyses are also very distinct. In summary, we have accurately obtained the morphological and phylogenetic characteristics of ten species.

Since Penzig [64] proposed the name *C. gloeosporioides*, numerous morphologically similar species have been grouped under the *C. gloeosporioides* complex [35]. Based on recent research, the *C*. *gloeosporioides* complex now comprises 51 closely related species [57]. A similar approach was used to distinguish other complexes, including *C. boninense* and *C. gigasporum* species complexes [37,38]. Most *C. gloeosporioides* isolates are associated with *Citrus* but also occur on other hosts, including *Carya* in Australia, *Mangifera indica* in South Africa, and *Pueraria* in America [35]. *C. kahawae*, belonging to the subclades of the *C. gloeosporioides* species complex, has been identified as a pathogen of Arabica coffee (*Coffea arabica*). Due to its unique adaptability to infect green berries, it occupies an ecological niche previously unoccupied by other fungi, leading to its classification as a novel species [35,59,65]. Studies have indicated that *C. kahawae* exhibits a certain level of host specificity; however, it has recently been identified as a pathogen for apples and grapes [60,66], suggesting host shift as the primary mechanism for the emergence of novel fungal pathogens [67]. However, when maize leaves were the host, *C. kahawae* was not the pathogenic agent, and no endophyte was obtained during the isolation and identification of healthy maize leaves. *C. brevisporum* was detected on *Neoregelia* sp. and *Pandanus pygmaeus* in Thailand, *Capsicum annuum* in China, and *Capsicum chinense* in Brazil [8,63,68]. *C. gigasporum* was first identified in the healthy leaves of *Centella asiatica* in Madagascar, *Stylosanth guianensis* in Mexico, and *Coffea arabica* in Colombia [37]. And recently, *C. gigasporum* was first identified as an etiological agent of litchi anthracnose [12]. These four strains (*C. gloeosporioides*, *C. kahawae*, *C. brevisporum,* and *C. gigasporum*) were isolated from leaves with typical maize leaf spot disease; however, they did not induce pathogenicity in maize leaves, and they were not proven to be endophytes in subsequent isolations of healthy maize leaves. Therefore, more healthy samples will be collected for verification.

*Colletotrichum cliviae* was first reported as a novel species from *Clivia miniata* leaves in China [31] and as an endophyte on *Camellia sinensis* and *Mangifera indica* in Brazil and China, respectively [7,33]. Recently, *C. cliviicola* was proposed as a new name for *C. cliviae* [38]. *C. cliviicola* has been identified as a pathogen of tobacco (*Nicotiana tabacum*), cowpea (*Vigna unguiculata* L. Walp), and persimmon [69,70,71]. *Colletotrichum fructicola* was described as a species associated with coffee berries, with a broad host range and wide geographical distribution, commonly identified as a pathogen of fruits such as blueberry, strawberry, and Litchi [5,10,11,12,31,35]. Recently, *C. karstii* was discovered on Orchidaceae in southwestern China and is recognized as the most common and geographically diverse species within the *C. boninense* complex, causing diseases across a broad range of hosts, including *Clivia miniata* and *Coffea* sp; *C. boninense* also exhibits a broad host range, but is more frequently identified as an endophyte [34,72]. *Colletotrichum siamense* was first discovered in coffee in Thailand [5] and has since been isolated from fruit rot (*Persea americana*) in Australia and leaf spot (*Hymenocallis americana*), pepper (*Capsicum annuum*) anthracnose, and Litchi (*Litchi chinensis*) anthracnose in China [8,12,35]. *C. truncatum* has numerous host species worldwide, with Fabaceae being the most common [31,62]. In this study, *C. cliviicola*, *C. fructicola*, *C. siamense*, *C. karstii*, and *C. truncatum* exhibited varying aggressiveness towards maize, while *C. boninense* was present as an endophyte. To our knowledge, we report maize for the first time as a host for these species.

*Colletotrichum* species are renowned for their intraspecific variability and broad host range [58]. Concurrently, we observed varying aggressiveness of different species within the same species complex, such as *C. karstii* and *C. boninense*. Significant variation in host specificity among species within a complex is consistent with the results reported by Liu et al. [33]. Our host specificity tests revealed that most strains are pathogenic to pears and apples, except that *C. boninense* could not infect apples, which is consistent with the results of Liu et al. [8]. Furthermore, five species (*C. siamense*, *C. fructicola*, *C. gloeosporioides*, *C. cliviicola*, and *C. karstii*) isolated from maize in our study were pathogenic to strawberry [11].

Different species of *Colletotrichum* have different lifestyles on various hosts, with some species primarily existing as endophytes and later switching to a necrotrophic mode of nutrition [73]. Numerous studies have indicated that the presence of wounds promotes infection by *Colletotrichum* species, such as mango fruit [74], chili fruit [75], and litchi leaves [12]. In the pathogenicity test, we found that when inoculating potted seedlings, the success rate of non-wounding inoculation was lower than that of wounding inoculation.

We found that *C. truncatum* can also cause spots on maize leaves regardless of wounding or not, although its field pathogenicity tests were unsuccessful. This indicates that appropriate environmental conditions have a significant impact on the infection of fungi [76]. This is why we have a low disease incidence in the field, even when using wounded inoculation. Temperature plays a significant role in the development of anthracnose by affecting mycelial growth, conidial germination, and appressorium development [77,78]. In our pathogenicity trials, *C. siamense*, which exhibited the fastest growth rate, also possessed a higher aggressivity, while among the other species with slower growth rates, only *C. karstii* was successful in wound inoculation with low success rates. Similarly, *C. cliviicola* was successful in causing disease under all three conditions, while, in contrast, *C. fructicola* failed in non-wounding inoculation. However, this is not always the case, as the pathogenicity of fungi is influenced by various factors, including environmental conditions such as humidity, as well as the host’s physical barriers like the cuticle, wax layer, secondary metabolites, and subsequent immune responses [76,77,78]. For instance, when inoculating tea plant leaves, *C. fructicola* showed greater adaptability than *C. camelliae*, but its aggressivity was weaker than that of *C. camelliae* [77].

Furthermore, these species have not previously been reported as pathogens of maize anthracnose in China. In our study, the strains of *Colletotrichum* isolated from healthy maize leaves belonged to *C. cliviicola*, *C. fructicola*, *C. karstii*, and *C. boninense*. The pathogenicity tests for these four strains revealed that only *C. boninense* was harmless to maize leaves. We believe that *C. boninense* is an endophyte in maize leaves. Endophytes have the potential to transition into pathogens under certain conditions, suggesting that maize anthracnose may occur naturally [79]. Although endophytes infect living plant tissues without causing disease symptoms, they have the potential to switch between endophytic and necrotrophic lifestyles over evolutionary and ecological timescales, playing an essential role in the life cycle of *Colletotrichum* species [7,80]. In the field, leaf damage, such as southern leaf blight, is common owing to weather conditions or diseases. We hypothesize that this is one of the factors that cause endophytic *Colletotrichum* species to become pathogens.

*Colletotrichum graminicola*, the common cause of leaf blight in maize, was not isolated in this study [6,15,16,18,20]. We speculate that *C. graminicola* may have lost its ecological niche on maize leaves in Sichuan. However, it is also possible that this species was missed during sampling or isolation.

In summary, this is the first report of *C. cliviicola*, *C. fructicola*, *C. siamense*, *C. karstii*, and *C. truncatum* as the causative agents of maize anthracnose in Sichuan. It is the first time these pathogens have been recognized as contributors to anthracnose in maize in China. In addition, *C. boninense* was recorded for the first time as an endophyte colonizing maize leaves in China. These findings significantly contribute to preventing and controlling maize leaf spot disease in Sichuan.

## 5. Conclusions

In this study, to ascertain the species of *Colletotrichum* associated with maize and their potential pathogenicity, we collected and isolated samples showing anthracnose symptoms. Utilizing cultural characteristics and phylogenetic analysis of the isolates, we successfully identified ten distinct species. Pathogenicity assays confirmed that species *C. cliviicola*, *C. fructicola*, *C. siamense*, *C. karstii*, and *C. truncatum* are the causative agents of maize. In contrast, species *C. boninense* exists as an endophyte within the maize. Our research has expanded the diversity of *Colletotrichum* species associated with maize in China and potentially worldwide, although *C. graminicola* was not identified in our study. This information provides a scientific foundation for the comprehensive prevention and management of maize leaf diseases and aids in developing resistant maize varieties.

## Figures and Tables

**Table 1:** Primers used for PCR amplification and sequencing for *Colletotrichum* spp.

Gene	Product Name	Primer	Primer Sequence (5′-3′)	Length (bp)	References
*GAPDH*	Glyceraldehyde-3-phosphate dehydrogenase	GDF	GCCGTCAACGACCCCTTCATTGA	200	[43]
		GDR	GGGTGGAGTCGTACTTGAGCATGT		
ITS	Internal transcribed spacer	ITS1	TCCGTAGGTGAACCTGCGG	550	[47,48]
		ITS4	TCCTCCGCTTATTGATATGC		
*TUB2*	β-tubulin	BT2a	GGTAACCAAATCGGTGCTGCTTTC	500	[45]
		BT2b	ACCCTCAGTGTAGTGACCCTTGGC		
*ACT*	Actin	ACT512F	ATGTGCAAGGCCGGTTTCGC	300	[44]
		ACT783R	TACGAGTCCTTCTGGCCCAT		
*CAL*	Calmodulin	CL1	GARTWCAAGGAGGCCTTCTC	688	[46]
		CL2	TTTTTGCATCATGAGTTGGAC		

**Table 2:** Summary of morphological data for *Colletotrichum* isolates.

Group ^x^	Species	Colonies’ Appearance	Growth Rate (mm/d) ^y^	Conidia			Conidial Appressorium			Mycelial Appressorium
				Length (μm)	Width (μm)	Shape	Width (μm)	Length (μm)	Characteristic	
1(32)	*C. gloeosporioides* *C. simense* *C. fructicola*	Sparse, white hyphae	5.9 ± 0.3 a ^z^	14.5 ± 1.5 f	5.0 ± 0.6 f	cylindrical	7.2 ± 0.6 f	5.9 ± 0.4 e	dark brown, oval to round	ovoid, light brown to dark brown, smooth edges
2(24)	*C. cliviicola*	Dense, pale gray with dark gray edge hyphae	5.5 ± 0.2 b	16.5 ± 1.0 e	5.6 ± 0.3 e	fusiform	9.1 ± 1.1 d	8.1 ± 0.8 b	dark brown, irregular with crenate edge	ovoid, light brown to brown with lobed
3(5)	*C. truncatum*	Sparse, white with yellowish hyphae	4.5 ± 0.4 e	26.6 ± 1.4 a	3.2 ± 0.2 g	falcate	8.6 ± 1.1 d	5.6 ± 0.6 e	brown, ovoid with smooth edge	ovoid, brown to dark brown, smooth edges
4(7)	*C. boninense*	Sparse, pale yellowish with white hyphae	4.9 ± 0.2 c	19.2 ± 1.4 d	8.4 ± 0.5 b	taper	9.3 ± 1.0 e	6.9 ± 1.3 d	dark brown, irregular with crenate edge	ovoid, brown with lobed or smooth edges
5(16)	*C. karstii*	Sparse, white hyphae	4.6 ± 0.2 de	14.4 ± 0.6 f	6.9 ± 0.2 c	cylindrical	11.1 ± 0.4 c	7.7 ± 1.1 bc	brown, ovoid with crenate edge	ovoid to irregular, light brown to dark brown, smooth edge
6(2)	*C. gigasporum*	Dense, white hyphae	4.7 ± 0.2 cd	25.0 ± 1.4 b	8.6 ± 0.4 a	long cylindrical	14.1 ± 0.6 a	8.7 ± 0.7 a	brown, ovoid with smooth edge	irregular ovoid brown to dark brown with slightly lobed edges
7(7)	*C. kahawae*	Dense, black hyphae with white edge hyphae	4.6 ± 0.4 de	14.3 ± 0.7 f	5.6 ± 0.3 e	cylindrical	11.9 ± 0.6 b	7.4 ± 1.4 c	brown, ovoid with crenate edge	ovoid, brown to dark brown with slightly lobed edges
8(6)	*C. brevisporum*	Sparse, gray to black with gray hyphae	4.8 ± 0.1 c	20.1 ± 1.4 c	5.8 ± 0.3 d	long cylindrical	8.6 ± 0.4 e	7.3 ± 0.5 c	dark brown, oval to round	ovoid to irregular, light to dark brown with smooth edges

^x^ The numbers shown in parentheses represent the number of isolates in each group. ^y^ Data are mean ± standard deviation. ^z^ Mean values with same letter in a column do not significantly differ (*p* > 0.05) according to Duncan’s multiple range test.

**Table 3:** The incidence caused by different species of *Colletotrichum* on variety Zhongyu 3 ^a^.

Species	Potted Maize Seedling ^b^		Maize Grown in the Field ^d^
	Wound ^b^	Non-Wound ^c^	
*C. cliviicola*	57.1	38.9	33.3
*C. fructicola*	68.0	0.0	40.0
*C. karstii*	54.2	0.0	16.7
*C. siamense*	86.4	72.2	53.3
*C. truncatum*	60.9	50.0	0
*C. boninense*	0	0	0
*C. kahawae*	0	0	0
*C. gigasporum*	0	0	0
*C. brevisporum*	0	0	0
*C. gloeosporioides*	0	0	0

^a^ The morbidities were recorded at 5 days after inoculation. ^b^ Suspension inoculation assessed leaf incidence at 18–27 locations on 9 seedlings. ^c^ Spraying inoculation was applied to 18 maize leaves on 9 seedlings. ^d^ Counting the incidence rate of 30 inoculation locations.

**Table 4:** Pathogenicity test of various *Colletotrichum* species on pear and apple ^x^.

Species	Isolate	Mean Lesion Diameter (cm)	
		Crown Pear	Red Fuji Apple
*Colletotrichum cliviicola*	YMTJ110	2.9 ± 0.5 b ^y^	1.3 ± 0.1 c
*C. fructicola*	YMTJ120	2.8 ± 0.7 b	2.4 ± 0.5 b
*C. karstii*	YMTJ118	0.7 ± 0.3 d	0.6 ± 0.1 d
*C. siamense*	YMTJ117	4.4 ± 0.7 a	3.5 ± 0.3 a
*C. truncatum*	YMTJ1	1.8 ± 0.9 c	0.5 ± 0.1 d
*C. boninense*	YMTJ116	0 e	0 e
*C. kahawae*	YMTJ27	0.8 ± 0.3 d	0 e
*C. brevisporum*	YMTJ58	2.5 ± 0.4 b	0 e
*C. gigasporum*	YMTJ12	2.2 ± 0.1 bc	0 e
*C. gloeosporioides*	YMTJ55	2.8 ± 0.3 b	0.8 ± 0.1 d
CK		0 e	0 e

^x^ Disease symptoms were recorded at 4 days after inoculation. ^y^ Data are mean ± standard deviation. The length and width indicated the diseased lesion. Mean values with same letter in a column do not significantly differ (*p* > 0.05) according to Duncan’s multiple range test.

## Data Availability

The data generated during this study are available on request from the corresponding author.

## Supplementary Materials

The following supporting information can be downloaded at https://www.mdpi.com/article/10.3390/jof10110799/s1: Table S1: *Colletotrichum* species isolated from maize leaves of Sichuan, P. R. China.; Table S2: Details of different *Colletotrichum* isolates used in this study; Table S3: *Colletotrichum* species in different years. Figure S1: Neighbor-joining trees based on ITS, *GAPDH*, *TUB*, *CAL*, and *ACT* genes of *Colletotrichum* isolates, respectively. Reference strains from this study are shown in red and bold. Figure S2: A Maximum likelihood tree of the *Colletotrichum* species. Phylogenetic relationships of *Colletotrichum* species associated with maize leaves from Sichuan Province, China, based on concatenated sequences of *ACT*, *TUB2*, *CAL*, *GAPDH*, and ITS genes, each analyzed with a separate model of DNA evolution. ML bootstrap and SH-aLRT support values were shown at the nodes (ML ≥ 60%). Isolates from this study are shown in red and bold. The tree is rooted with *Monilochaetes infuscans*. Figure S3: Maximum parsimony phylogenetic analysis of the 48 representative strains obtained from maize leaf spots in Sichuan Province, China. The tree obtained with the concatenated sequences of *ACT*, *TUB2*, *CAL*, *GAPDH*, and ITS. Parsimony bootstrap values of more than 60% are shown at the nodes. The tree is rooted with *Monilochaetes infuscans*.
