# Supplementary material for: Identification and Characterization of Colletotrichum Species Associated with Maize in Sichuan, China
Source: J Fungi (Basel). 2024 Nov 18;10(11):799. doi: 10.3390/jof10110799 (PMC11595826; doi:10.3390/jof10110799)
Supplement: Supplementary file 1 [file jof-10-00799-s001.zip › jof-3302549-supplementary_tables.pdf]

Journal of Fungi

MDPI

Supplementary Materials

Table S1. *Colletotrichum* species isolated from maize leaves of Sichuan, P. R. China.

Species	Isolate No.	Origin	Accession No. (GAPDH)	Morphological Group
C. truncatum	YMTJ1	Lushan, Yaan	MK569076	Group3
C. cliviicola	YMTJ2	Weiyuan, Neijiang	MK569051	Group2
C. truncatum	YMTJ3	Lushan, Yaan	MK569077	Group3
C. kahawae	YMTJ4	Baoxing, Yaan	MK569098	Group7
C. siamense	YMTJ5	Weiyuan, Neijiang	MK569067	Group1
C. fructicola	YMTJ6	Renshou, Meishan	MK544866	Group1
C. siamense	YMTJ7	Weiyuan, Neijiang	MK569068	Group1
C. fructicola	YMTJ8	Renshou, Meishan	MK544867	Group1
C. siamense	YMTJ9	Weiyuan, Neijiang	MK569069	Group1
C. karstii	YMTJ10	Jianyang, Chengdu	MK569060	Group5
C. siamense	YMTJ11	Zhongjiang, Deyang	MK569070	Group1
C. gigasporum	YMTJ12	Baoxing, Yaan	MK569096	Group6
C. fructicola	YMTJ13	Weiyuan, Neijiang	MK544868	Group1
C. cliviicola	YMTJ14	Xichang, Liangshan	MK569052	Group2
C. karstii	YMTJ15	Zhongjiang, Deyang	MK569061	Group5
C. fructicola	YMTJ16	Weiyuan, Neijiang	MK544869	Group1
C. siamense	YMTJ17	Lushan, Yaan	MK569071	Group1
C. siamense	YMTJ18	Zhongjiang, Deyang	MK569072	Group1
C. truncatum	YMTJ20	Zhongjiang, Deyang	MK569117	Group3
C. truncatum	YMTJ21	Zhongjiang, Deyang	MK569118	Group3
C. brevisporum	YMTJ22	Zhongjiang, Deyang	MK569120	Group8
C. fructicola	YMTJ25	Zhongjiang, Deyang	MK544870	Group1
C. brevisporum	YMTJ26	Zhongjiang, Deyang	MK569121	Group8
C. kahawae	YMTJ27	Zhongjiang, Deyang	MK569099	Group7
C. kahawae	YMTJ28	Zhongjiang, Deyang	MK569100	Group7
C. cliviicola	YMTJ30	Jianyang, Chengdu	MK569080	Group2
C. cliviicola	YMTJ31	Jianyang, Chengdu	MK569081	Group2
C. fructicola	YMTJ33	Jianyang, Chengdu	MK544871	Group1
C. cliviicola	YMTJ34	Jianyang, Chengdu	MK569082	Group2
C. cliviicola	YMTJ35	Tianquan, Yaan	MK569083	Group2
C. siamense	YMTJ39	Zhongjiang, Deyang	MK569113	Group1
C. boninense	YMTJ40	Lushan, Yaan	MK544884	Group4
C. karstii	YMTJ41	Renshou, Meishan	MK569104	Group5
C. cliviicola	YMTJ42	Jianyang, Chengdu	MK569084	Group2
C. truncatum	YMTJ43	Renshou, Meishan	MK569119	Group3
C. cliviicola	YMTJ45	Zhongjiang, Deyang	MK569085	Group2
C. brevisporum	YMTJ46	Renshou, Meishan	MK569122	Group8
C. karstii	YMTJ47	Cuiping, Yibin	MK569105	Group5
C. siamense	YMTJ48	Jianyang, Chengdu	MK569114	Group1
C. cliviicola	YMTJ49	Rongxian, Zigong	MK569086	Group2
C. kahawae	YMTJ51	Jianyang, Chengdu	MK569101	Group7
C. siamense	YMTJ52	Tianquan, Yaan	MK569115	Group1
C. cliviicola	YMTJ53	Luding, Ganzi	MK569087	Group2
C. cliviicola	YMTJ54	Luding, Ganzi	MK569088	Group2

C. gloeosporioides	YMTJ55	Renshou, Meishan	MK569079	Group1
C. boninense	YMTJ56	Tianquan, Yaan	MK544885	Group4
C. karstii	YMTJ57	Renshou, Meishan	MK569106	Group5
C. brevisporum	YMTJ58	Rongxian, Zigong	MK569123	Group8
C. cliviicola	YMTJ61	Tianquan, Yaan	MK569089	Group2
C. boninense	YMTJ64	Yucheng, Yaan	MK544886	Group4
C. gigasporum	YMTJ66	Yucheng, Yaan	MK569097	Group6
C. karstii	YMTJ67	Yucheng, Yaan	MK569107	Group5
C. fructicola	YMTJ68	Dayi, Chengdu	MK544872	Group1
C. cliviicola	YMTJ69	Yucheng, Yaan	MK569090	Group2
C. fructicola	YMTJ70	Cuiping, Yibin	MK544873	Group1
C. fructicola	YMTJ71	Cuiping, Yibin	MK544874	Group1
C. karstii	YMTJ72	Xingdu, Chengdu	MK569062	Group5
C. siamense	YMTJ73	Zhongjiang, Deyang	MK569116	Group1
C. karstii	YMTJ74	Zhongjiang, Deyang	MK569063	Group5
C. karstii	YMTJ75	Cuiping, Yibin	MK569064	Group5
C. karstii	YMTJ76	Renshou, Meishan	MK569108	Group5
C. karstii	YMTJ77	Renshou, Meishan	MK569065	Group5
C. brevisporum	YMTJ78	Jianyang, Chengdu	MK569124	Group8
C. cliviicola	YMTJ81	Zhongjiang, Deyang	MK569053	Group2
C. cliviicola	YMTJ84	Yucheng, Yaan	MK569091	Group2
C. kahawae	YMTJ85	Yucheng, Yaan	MK569102	Group7
C. boninense	YMTJ86	Dayi, Chengdu	MK544887	Group4
C. karstii	YMTJ87	Dayi, Chengdu	MK569109	Group5
C. karstii	YMTJ88	Dayi, Chengdu	MK569110	Group5
C. karstii	YMTJ89	Yucheng, Yaan	MK569111	Group5
C. cliviicola	YMTJ90	Chongzhou, Chengdu	MK569092	Group2
C. karstii	YMTJ91	Yucheng, Yaan	MK569112	Group5
C. fructicola	YMTJ95	Dayi, Chengdu	MK544875	Group1
C. fructicola	YMTJ96	Dayi, Chengdu	MK544876	Group1
C. fructicola	YMTJ97	Qionglai, Chengdu	MK544877	Group1
C. fructicola	YMTJ98	Qionglai, Chengdu	MK544878	Group1
C. cliviicola	YMTJ99	Qionglai, Chengdu	MK569093	Group2
C. kahawae	YMTJ104	Lushan, Yaan	MK569103	Group7
C. fructicola	YMTJ105	Mabian, Leshan	MK544879	Group1
C. boninense	YMTJ106	Yucheng, Yaan	MK544888	Group4
C. cliviicola	YMTJ107	Lushan, Yaan	MK569094	Group2
C. cliviicola	YMTJ108	Mabian, Leshan	MK569054	Group2
C. cliviicola	YMTJ109	Mabian, Leshan	MK569095	Group2
C. cliviicola	YMTJ110	Chongzhou, Chengdu	MK569055	Group2
C. siamense	YMTJ111	Renhe, Panzhihua	MK569073	Group1
C. kahawae	YMTJ113	Yucheng, Yaan	MK569059	Group7
C. siamense	YMTJ114	Renhe, Panzhihua	MK569074	Group1
C. cliviicola	YMTJ115	Chongzhou, Chengdu	MK569056	Group2
C. boninense	YMTJ116	Lushan, Yaan	MK544889	Group4
C. siamense	YMTJ117	Yucheng, Yaan	MK569075	Group1
C. karstii	YMTJ118	Chongzhou, Chengdu	MK569066	Group5
C. cliviicola	YMTJ119	Chongzhou, Chengdu	MK569057	Group2
C. fructicola	YMTJ120	Chongzhou, Chengdu	MK544880	Group1
C. brevisporum	YMTJ121	Zhongjiang, Deyang	MK569078	Group8
C. fructicola	YMTJ122	Zhongjiang, Deyang	MK544881	Group1

C. fructicola	YMTJ123	Zhongjiang, Deyang	MK544882	Group1
C. fructicola	YMTJ124	Zhongjiang, Deyang	MK544883	Group1
C. cliviicola	YMTJ125	Youxian, Mianyang	MK569058	Group2
C. boninense	YMTJ126	Beichuan, Mianyang	MK544890	Group4

Table S2. Details of different *Colletotrichum* isolates used in this study [x].

Species	Isolate Number	Host	Location	GenBank Accession Numbers				
				ACT	*CAL*	*GAPDH*	ITS	*TUB2*
C. boninense	**YMTJ40** [x]	*Zea Mays*	China	—	MK569125	MK544884	MK569220	MK569174
	**YMTJ56**	*Zea Mays*	China	—	MK569126	MK544885	MK569221	MK569175
	**YMTJ64**	*Zea Mays*	China	—	—	MK544886	MK569222	MK569176
	**YMTJ116**	*Zea Mays*	China	—	MK569127	MK544889	MK569224	MK569177
	CBS 128547, ICMP 10338 [y]	*Camellia* sp.	New Zealand	JQ005507	JQ005680	JQ005246	JQ005159	JQ005593
	CBS 112115, STE-U 2966	*Leucospermum sp.*	Australia	JQ005501	JQ005674	JQ005247	JQ005160	JQ005588
	CBS 123755, MAFF 305972*[z]	*Crinum asiaticum* var. *sinicum*	Japan	HM582001	HM582004	HM585386	HM585399	HM585421
	CSSN1	*Crinum asiaticum*	China	GQ856774	GQ849462	GQ856743	GQ485597	GQ849437
	CSSX8	*Crinum asiaticum*	China	GQ856771	GQ849460	GQ856742	GQ485596	GQ849433
C. brevisporum	**YMTJ58**	*Zea Mays*	China	MK602880	MK569128	MK569123	MK569226	—
	**YMTJ78**	*Zea Mays*	China	MK602881	MK569129	MK569124	MK569227	—
	LJTJ24	*Capsicum* sp.	China	KP823736	—	KP823794	KP748215	—
	LJTJ54	*Capsicum sp.*	China	KP943568	—	KP943511	KP943578	—
	BCC 38876*	*Neoregalia sp.*	Thailand	JN050216	JN050222	JN050227	JN050238	JN050244
C. cliviicola	**YMTJ2**	*Zea Mays*	China	MK602882	MK569130	MK569051	MK569244	MK569178
	**YMTJ14**	*Zea Mays*	China	MK602883	MK569131	MK569052	MK569245	MK569179
	**YMTJ30**	*Zea Mays*	China	MK602884	MK569132	MK569080	MK569246	MK569180
	**YMTJ31**	*Zea Mays*	China	MK602885	MK569133	MK569081	MK569247	MK569181
	**YMTJ35**	*Zea Mays*	China	MK602886	MK569134	MK569083	—	MK569182
	**YMTJ42**	*Zea Mays*	China	MK602887	MK569135	MK569084	MK569248	MK569183
	**YMTJ45**	*Zea Mays*	China	MK602888	MK569136	MK569085	MK569249	MK569184
	**YMTJ110**	*Zea Mays*	China	MK602889	MK569137	MK569055	MK569255	MK569185
	**YMTJ115**	*Zea Mays*	China	MK602890	MK569138	MK569056	MK569256	MK569186
	**YMTJ119**	*Zea Mays*	China	MK602891	MK569139	MK569057	MK569257	MK569187
	CBS 125375*	*Clivia miniata*	China	JX519240	—	JX546611	JX519223	JX519249
	CSSK4	*Clivia miniata*	China	GQ856777	GQ849464	GQ856756	GQ485607	GQ849440
	CSSS1	*Clivia miniata*	China	GU085861	GU085864	GU085868	GU109479	GU085869
	CORCG2	*Cymbidium hookerianum*	China	HM581985	HM582007	HM585380	HM585397	HM585422
	CORCX9	*Arundina graminifolia*	China	HM581986	HM582008	HM585381	HM585398	HM585423
C. fructicola	**YMTJ6**	*Zea Mays*	China	MK602892	MK569140	MK544866	MK569258	MK569188
	**YMTJ13**	*Zea Mays*	China	—	MK569141	MK544868	MK569260	MK569189
	**YMTJ16**	*Zea Mays*	China	MK602893	MK569142	MK544869	MK569261	MK569190

	**YMTJ25**	*Zea Mays*	China	MK602894	MK569143	MK544870	MK569262	MK569191
	**YMTJ33**	*Zea Mays*	China	MK602895	MK569144	MK544871	MK569263	MK569192
	**YMTJ48**	*Zea Mays*	China	MK602913	MK569165	MK569114	—	MK569212
	**YMTJ120**	*Zea Mays*	China	MK602896	MK569145	MK544880	MK569268	MK569193
	MFLUCC090228*	*Coffea arabica*	Thailand	FJ907426	FJ917508	FJ972578	FJ972603	FJ907441
	CBS 238.49*, ICMP 17921	*Ficus habrophylla*	Germany	JX009495	JX009671	JX009923	JX010181	JX010400
	CBS 125397*, ICMP 18646	*Tetragastris panamensis*	Panama	JX009581	JX009674	JX010032	JX010173	JX010409
	LJTJ2	*Capsicum* sp.	China	KP823742	KP823812	KP823772	KP748192	KP823854
	LJTJ10	*Capsicum* sp.	China	KP823743	KP823813	KP823780	KP748201	KP823855
C. gigasporum	**YMTJ12**	*Zea Mays*	China	MK602897	MK569146	MK569096	MK569269	MK569194
	**YMTJ66**	*Zea Mays*	China	MK602898	MK569147	MK569097	MK569270	MK569195
	CBS101881	*Solanum betaceum*	New Zealand	KF687797	KF687808	KF687841	KF687736	KF687886
	CBS133266, MUCL44947*	*Centella asiatica*	Madagascar	—	—	KF687822	KF687715	KF687866
C. gloeosporioides	**YMTJ55**	*Zea Mays*	China	MK602899	MK569148	MK569079	MK569271	MK569196
	CORCG5	*Vanda* sp.	China	HM034801	HM034803	HM034807	HM034809	HM034811
	IMI 356878*, ICMP 17821	*Citrus sinensis*	Italy	JX009531	JX009731	JX010056	JX010152	JX010445
	LJTJ13	*Capsicum* sp.	China	KP823751	KP823821	KP823783	KP748204	KP823863
C. kahawae	**YMTJ4**	*Zea Mays*	China	MK602900	MK569149	MK569098	MK569272	MK569197
	**YMTJ27**	*Zea Mays*	China	—	MK569150	MK569099	MK569273	MK569198
	**YMTJ28**	*Zea Mays*	China	MK602901	MK569151	MK569100	—	MK569199
	**YMTJ85**	*Zea Mays*	China	—	MK569152	MK569102	MK569275	MK569200
	**YMTJ104**	*Zea Mays*	China	—	MK569153	MK569103	MK569276	MK569201
	**YMTJ113**	*Zea Mays*	China	MK602902	MK569154	MK569059	MK569277	MK569202
	ICMP 12952	*Persea americana*	New Zealand	JX009431	JX009648	JX009971	HM542572	JX010426
	IMI 319418*, ICMP 17816	*Coffea arabica*	Kenya	JX009452	JX009642	JX010012	JX010231	JX010444
	ICMP 18539*	*Olea europaea*	Australia	JX009523	JX009635	JX009966	JX010230	JX010434
C. karstii	**YMTJ10**	*Zea Mays*	China	MK602903	MK569155	MK569060	MK569278	MK569203
	**YMTJ41**	*Zea Mays*	China	MK602904	MK569156	MK569104	MK569280	MK569204
	**YMTJ67**	*Zea Mays*	China	MK602905	MK569157	MK569107	MK569282	MK569205
	**YMTJ76**	*Zea Mays*	China	MK602906	MK569158	MK569108	MK569283	—
	**YMTJ88**	*Zea Mays*	China	MK602907	MK569159	MK569110	MK569285	MK569206
	**YMTJ118**	*Zea Mays*	China	MK602908	MK569160	MK569066	MK569287	MK569207
	CBS13234, CGMCC 3.14194* CORCK6	*Vanda* sp.	China	HM581995	HM582013	HM585391	HM585409	HM585428
	CORCK1	*Calanthe argenteo-striata*	China	HM581991	HM582010	HM585387	HM585406	HM585424
	CORCK3	*Eria coronaria*	China	HM581992	HM582011	HM585388	HM585407	HM585427

	CORCS4	*Pleione bulbocodioides*	China	HM581994	HM582012	HM585390	HM585405	HM585426
	CORCX7	*Arundina graminifolia*	China	HM581993	HM582009	HM585389	HM585408	HM585425
C. siamense	**YMTJ5**	*Zea Mays*	China	MK602909	MK569161	MK569067	MK569232	MK569208
	**YMTJ9**	*Zea Mays*	China	MK602910	MK569162	MK569069	MK569234	MK569209
	**YMTJ18**	*Zea Mays*	China	MK602911	MK569163	MK569072	MK569237	MK569210
	**YMTJ39**	*Zea Mays*	China	MK602912	MK569164	MK569113	MK569238	MK569211
	**YMTJ52**	*Zea Mays*	China	—	MK569166	MK569115	MK569239	MK569213
	**YMTJ111**	*Zea Mays*	China	MK602914	MK569167	MK569073	MK569241	MK569214
	**YMTJ117**	*Zea Mays*	China	MK602916	MK569169	MK569075	MK569243	MK569215
	ICMP 17795	*Malus* x *domestica*	USA	JX009506	JX009703	JX010051	JX010162	JX010393
	CBS 130417, ICMP 18578*	*Coffea arabica*	Thailand	JX009518	JX009714	JX009924	JX010171	JX010404
	CBS 125378*, ICMP 18642	*Hymenocallis americana*	China	GQ856775	JX009709	JX010019	JX010278	JX010410
	LJTJ7	*Capsicum* sp.	China	KP823757	KP823826	KP823777	KP748198	KP823869
	LJTJ8	*Capsicum* sp.	China	KP823758	KP823827	KP823778	KP748199	KP823870
C. truncatum	**YMTJ1**	*Zea Mays*	China	MK602917	MK569170	MK569076	MK569228	MK569216
	**YMTJ3**	*Zea Mays*	China	MK602918	MK569171	MK569077	MK569229	MK569217
	**YMTJ20**	*Zea Mays*	China	MK602919	MK569172	MK569117	MK569230	MK569218
	**YMTJ21**	*Zea Mays*	China	MK602920	MK569173	MK569118	—	MK569219
	CBS 120709	*Capsicum frutescens*	India	GQ856783	GQ849453	GQ856753	GQ485593	GQ849429
	CBS 151.35*	*Phaseolus lunatus*	USA	GU227960	—	GU228254	GU227862	GU228156
	LJTJ1	*Capsicum* sp.	China	KP823762	KP823831	KP823771	KP748196	KP823840
Monilochaetes infuscans	CBS 869.96	*Ipomoea batatas*	South Africa	JQ005843	—	JX546612	JQ005780	JQ005864

x Strains in bold are from the current study. y BCC: BIOTEC Culture Collection, Thailand; CBS: Culture collection of the Westerdijk Fungal Biodiversity Institute, Utrecht, The Netherlands; CGMCC: China General Microbiological Culture Collection Center, China; ICMP: International Collection of Microorganisms from Plants, Auckland, New Zealand; IMI: Culture collection of CABI Europe UK Centre, Egham, UK; MAFF: MAFF Genebank Project, Ministry of Agriculture, Forestry and Fisheries, Tsukuba, Japan; MFLUCC: Mae Fah Luang University Culture Collection, Chiang Rai, Thailand; MUCL: BCCM/MUCL collection, Université catholique de Louvain, Belgium; STE-U: Culture collection of the Department of Plant Pathology, University of Stellenbosch, South Africa; * Ex-holotype or ex-epitype cultures. >C.boninense CSSN1 ttacccctccaggtcgtcatgatatcaagcccgccaacccggccaac-cgcccttcgccgcaggagcctggcagccaacggacacgagttcccagacaccgccagtggccgagatagtggg atgtgatactcgttt-ggctcaacaaagcttccaagccactcgctgactcgccctccgcaggcctacatgctcaagtacgactccacc.

Table S3. *Colletotrichum* species in different years.

Time	Target	Period of Maize	Samples	Numbers of *Colletotrichum*	Seperation Dpecies
2015.7-9	Leaf spot	Ripening stage	1060	100	*C. cliviicola, C. siamense, C. kahawae, C. boninense, C. karstii, C. fructicola, C. brevisporum, C. gigasporum, C. truncatum, C. gloeosporioides*

2017.7-9	Suspected anthracnose symptoms	Ripening stage	104	31	*C. cliviicola, C. siamense, C. kahawae, C. boninense, C. karstii, C. fructicola, C. brevisporum*
	Healthy leaves	Ripening stage	20	3	—
2018.5	Healthy leaves	Six leaf stage	100	14	*C. cliviicola, C. fructicola, C. karstii, C. boninense*
