# Supplementary material for: Identification and Characterization of Colletotrichum Species Associated with Maize in Sichuan, China
Source: J Fungi (Basel). 2024 Nov 18;10(11):799. doi: 10.3390/jof10110799 (PMC11595826; doi:10.3390/jof10110799)
Supplement: Supplementary file 1 [file jof-10-00799-s001.zip › Figure S1.pdf]

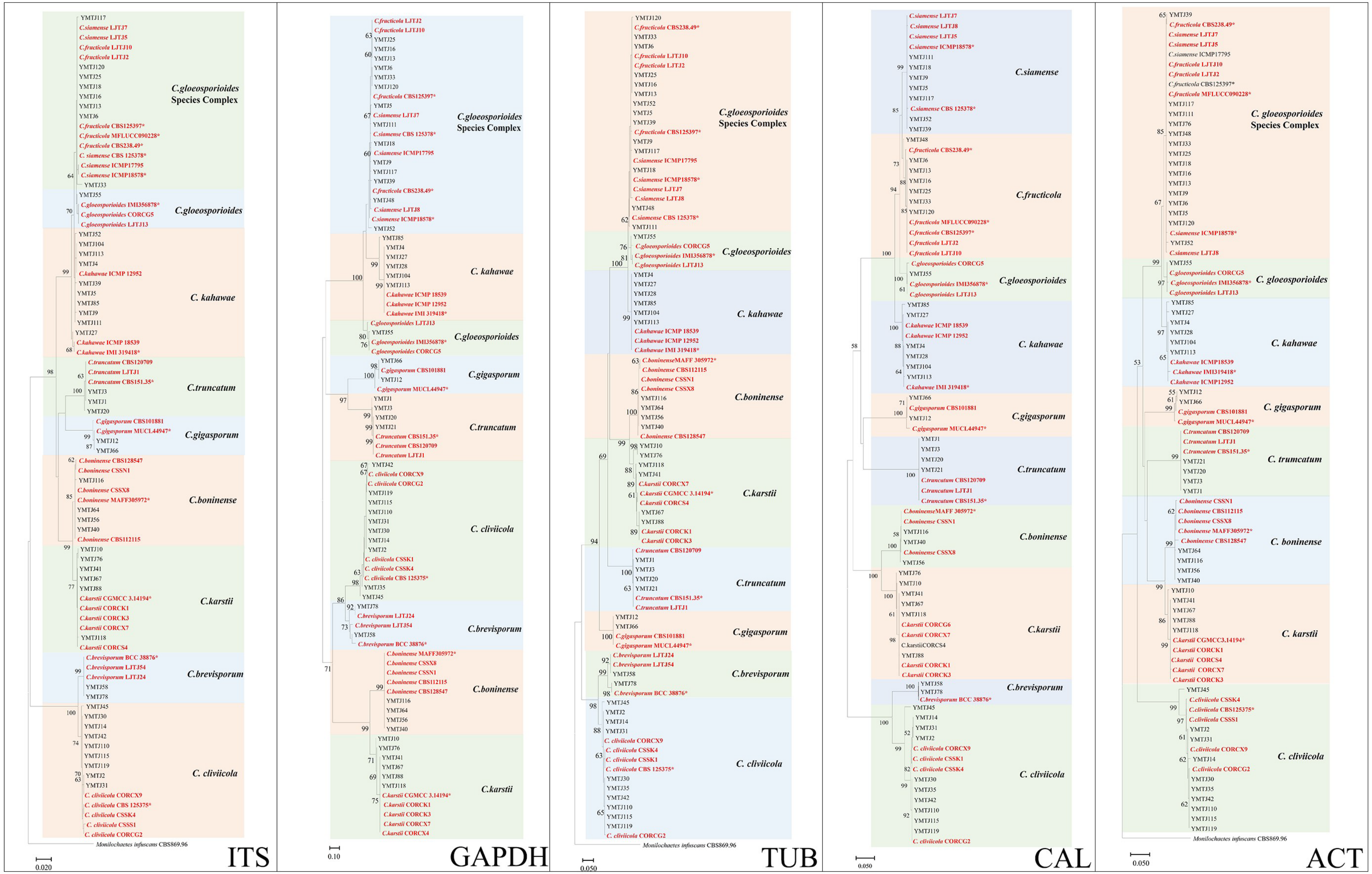

Figure. S1 Neighbor-Joining trees based on ITS, *GAPDH*, *TUB*, *CAL*, and *ACT* genes of *Colletotrichum* isolates, respectively. Reference strains from this study are shown in red and bold.
