# Supplementary material for: Identification and Characterization of Colletotrichum Species Associated with Maize in Sichuan, China
Source: J Fungi (Basel). 2024 Nov 18;10(11):799. doi: 10.3390/jof10110799 (PMC11595826; doi:10.3390/jof10110799)
Supplement: Supplementary file 1 [file jof-10-00799-s001.zip › Figure S2.pdf]

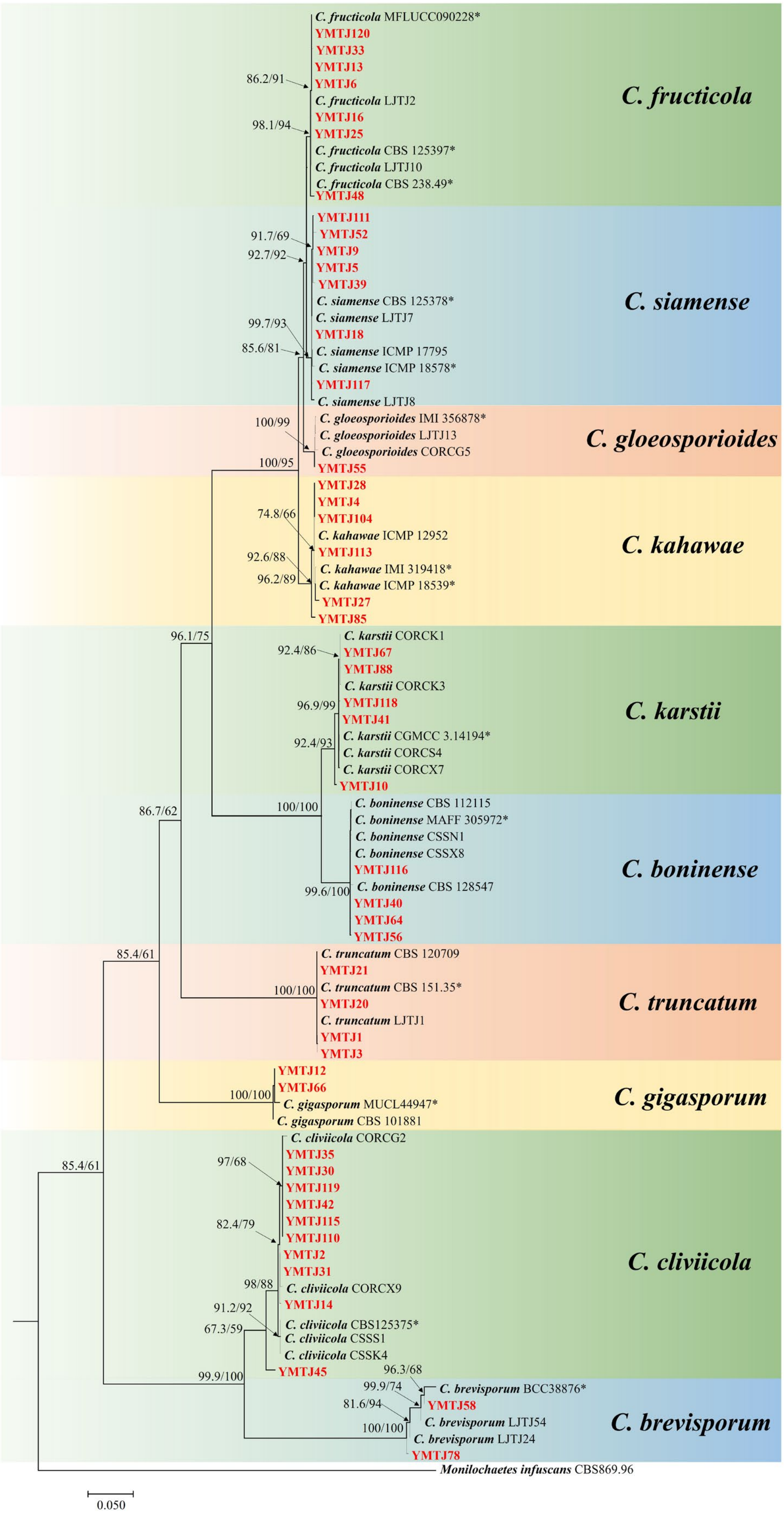

Figure. S2 A Maximum likelihood tree of the *Colletotrichum* species. Phylogenetic relationships of *Colletotrichum* species associated with maize leaves from Sichuan Province, China, based on concatenated sequences of *ACT*, *TUB2*, *CAL*, *GAPDH*, and ITS genes, each analyzed with a separate model of DNA evolution. ML bootstrap and SH-aLRT support values were shown at the nodes (ML ≥ 60%). Isolates from this study are shown in rea and bold. The tree is rooted with *Monilochaetes infuscans*.
