# Supplementary material for: Identification and Characterization of Colletotrichum Species Associated with Maize in Sichuan, China
Source: J Fungi (Basel). 2024 Nov 18;10(11):799. doi: 10.3390/jof10110799 (PMC11595826; doi:10.3390/jof10110799)
Supplement: Supplementary file 1 [file jof-10-00799-s001.zip › Figure S3.pdf]

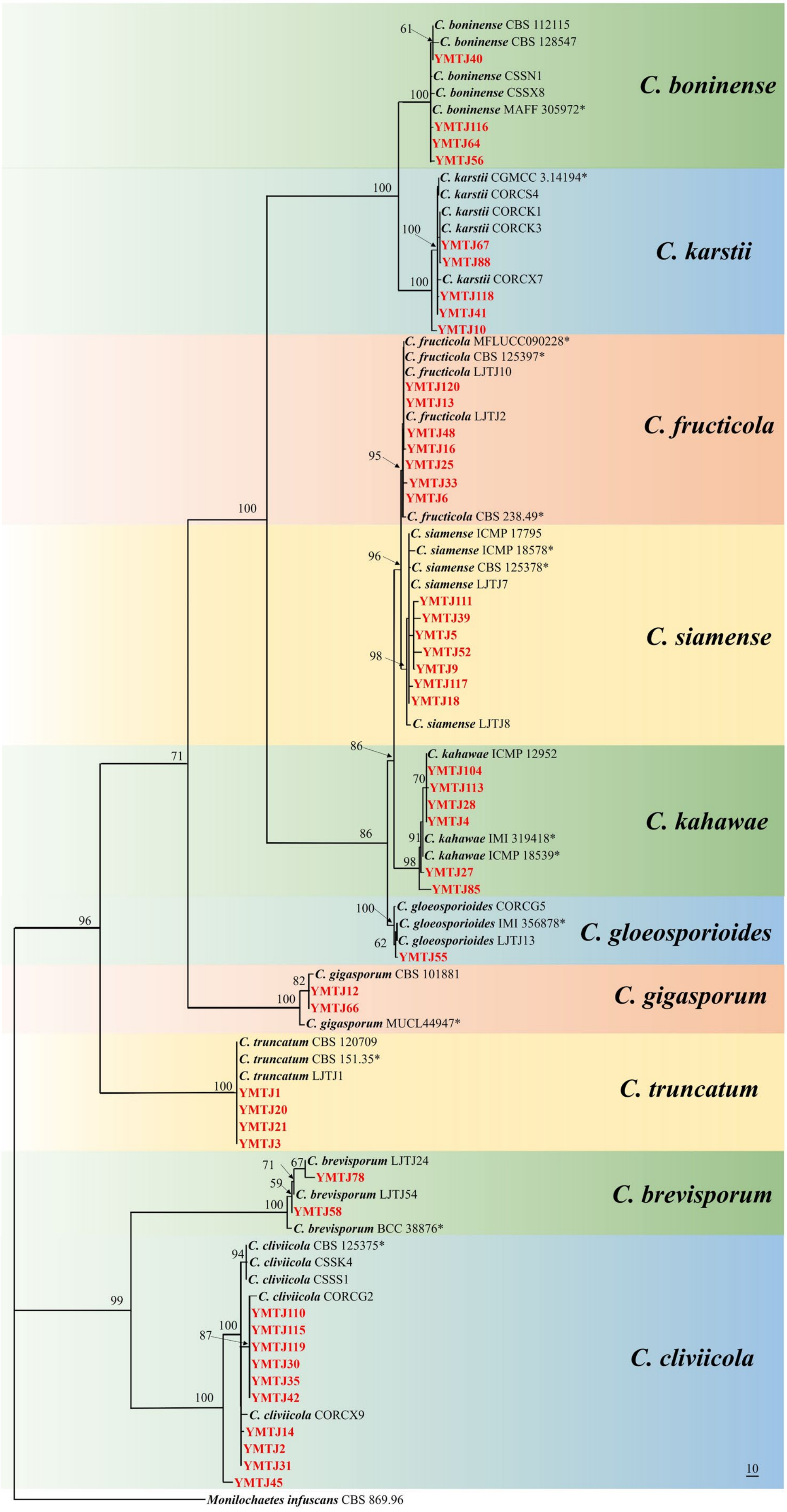

Figure. S3 Maximum parsimony phylogenetic analysis of the 48 representative strains associated with maize leaves from Sichuan Province, China. The tree conducted with the concatenated sequences of *ACT*, *TUB2*, *CAL*, *GAPDH*, and ITS. Parsimony bootstrap values of more than 60% are shown at the nodes. The tree is rooted with *Monilochaetes infuscans*.
